# Proteomics‐based screening of the target proteins associated with antidepressant‐like effect and mechanism of Saikosaponin A

**DOI:** 10.1111/jcmm.14695

**Published:** 2019-11-24

**Authors:** Juanjuan Guo, Feng Zhang, Jifang Gao, Xinyuan Guan, Beiyun Liu, Xiaoge Wang, Zhaoyu Qin, Kuanxiao Tang, Shilian Liu

**Affiliations:** ^1^ Department of Biochemistry and Molecular Biology School of Medicine Shandong University Jinan China; ^2^ School of Life Sciences Qilu Normal University Jinan China; ^3^ Department of Geriatrics Xuecheng People's Hospital Zaozhuang China; ^4^ Department of Pathology Linyi People’s Hospital Linyi China; ^5^ Bureau of Emergency Management of Siping City Siping China; ^6^ Shanghai United Imaging Healthcare Co., Ltd. Shanghai China; ^7^ Guangzhou Hospital of integrated Traditional and West Medicine Guangzhou China; ^8^ Laboratory of Systems Biology Institute of Biomedical Sciences Fudan University Shanghai China; ^9^ Department of Geriatrics Qilu Hospital of Shandong Univeristy Jinan China

**Keywords:** depression, dopamine, proline‐rich transmembrane protein 2, proteomics, Saikosaponin A

## Abstract

Depression is a commonly occurring neuropsychiatric disease with an increasing incidence rate. Saikosaponin A (SA), a major bioactive component extracted from Radix Bupleuri, possesses anti‐malignant cell proliferation, anti‐inflammation, anti‐oxidation and liver protective effects. However, few studies have investigated SA’s antidepressant effects and pharmacological mechanisms of action. Our study aimed to explore the anti‐depression effect of SA and screen the target proteins regulated by SA in a rat model of chronic unpredictable mild stress (CUMS)‐induced depression. Results showed that 8‐week CUMS combined with separation could successfully produce depressive‐like behaviours and cause a decrease of dopamine (DA) in rat hippocampus, and 4‐week administration of SA could relieve CUMS rats’ depressive symptoms and up‐regulated DA content. There were 15 kinds of significant differentially expressed proteins that were detected not only between the control and CUMS groups, but also between the CUMS and SA treatment groups. Proline‐rich transmembrane protein 2 (PRRT2) was down‐regulated by CUMS while up‐regulated by SA. These findings reveal that SA may exert antidepressant effects by up‐regulating the expression level of PRRT2 and increasing DA content in hippocampus. The identification of these 15 differentially expressed proteins, including PRRT2, provides further insight into the treatment mechanism of SA for depression.

## 1 INTRODUCTION

With increasing stress factors, depression has become a commonly occurring and life‐threatening neuropsychiatric disease in modern society.^1^ With its high prevalence, recurrence, and enormous personal and societal costs, depression has attracted the attention of scientists all over the world.^2^, ^3^ Scholars around the globe have been devoting themselves to exploring the pathogenesis of depression in an attempt to find more effective and safer antidepressants.

A number of theories about the aetiology and course of depression have been proposed. In the 1960s, the classical monoamine hypothesis of depression was first proposed, stating that depression is due to a deficiency in one or more of three biogenic monoamines (dopamine [DA], 5‐hydroxytryptamine [5‐HT] and norepinephrine [NE]) in the central nervous system.^4^ Later, the hypotheses of neuroendocrinology 5, 6 and neuroplasticity 7 and the theory of inflammation and immunity 8 on depression were put forward successively. According to these theories, a good amount of scientific research has been performed to investigate the pathophysiological changes of depression. Thus, it can be seen that the pathogenesis of depression is complicated, involving several systems, multiple proteins and a lot of small molecules. No single theory or signal pathway can fully elucidate its pathogenesis. Therefore, we should consider all factors and pay attention to the interaction among them. Only in this way can we comprehensively and systematically elucidate the pathogenesis of depression.

In the past few years, proteomic technologies have been extensively applied to identify biomarkers, characterize complex biochemical systems and examine pathophysiological processes in various diseases.^9^ However, there are still no proteomic data available on the therapy mechanism of Saikosaponin A (SA) on stress‐induced depression. Therefore, in our study a quantitative proteomics technology—iTRAQ—was used to screen the differentially expressed target proteins regulated by chronic unpredictable mild stress (CUMS) and SA. The numerous differentially expressed proteins detected by iTRAQ and the bioinformatics analysis of them will help to enhance our understanding of the pathogenesis of depression and the mechanisms behind the antidepressant effects of SA.

SA, a major bioactive component isolated from Radix Bupleuri, is one kind of triterpene saponins, containing a sugar side chain of monodesmosides in an oleanane‐type triterpene skeleton at C‐3.^10^ This saponin exhibits a wide variety of pharmacological activities, causing anti‐malignant cell proliferation, anti‐inflammation, anti‐oxidation and liver protective effects.^11^, ^12^ Increasing studies have confirmed that SA has certain anti‐tumour effects through inducing cell apoptosis. It is reported that SA triggers caspase‐3 dependent and independent apoptosis mediated through the regulation of the Bcl‐2 family, leading to mitochondrial dysfunction and release of apoptotic factors in hepatic stellate cells.^10^ Ming Feng Chen and his team have demonstrated that Saikosaponin A and Saikosaponin D could induce cell apoptosis by inhibiting proliferation and migratory activity of rat HSC‐T6 cells.^13^ It is found SA induces caspase‐mediated apoptosis in human colon carcinoma (HCC) cells by triggering caspase‐2 and caspase‐8 activation, suggesting SA may be a promising cancer therapy agent in certain types of cancer.^14^ In recent years, Radix Bupleuri and saikosaponins have been demonstrated to possess antidepressant effects in in vivo and in vitro experiments.^15^, ^16^, ^17^, ^18^ A study published in Neuroscience Letters in 2017 demonstrates that administration of saikosaponin A for 4 weeks could produce the antidepressant‐like effects in perimenopausal rats, and the potential mechanism may be the restoration of neuroendocrine, neuroinflammation and neurotrophic systems in the hippocampus during perimenopausal.^19^ These results indicate that there is a strong correlation between SA and depression, but the exact therapeutic mechanisms of SA on depression need to be further explored.

A CUMS depression animal model has been widely used for investigating the pathophysiological mechanisms underlying depression and evaluating the efficacy of antidepressants.^20^ This stress‐induced model of depression has good validity and reliability.^21^ Moreover, it can overcome the stress habituation that usually occurs with chronic restricted stress (CRS) models and induce consolidated long‐lasting behavioural deficits.^22^

Therefore, in the present study, we utilized the CUMS depression model to investigate whether long‐term treatment with SA could prevent the CUMS‐induced depressive‐like behaviours and reverse monoamine neurotransmitter changes in the hippocampus. To further explore the underlying therapeutic mechanism of SA, iTRAQ was used to screen for differentially expressed proteins before and after CUMS (and SA) treatment. With reference to domestic and foreign literatures, we chose protein Proline‐rich transmembrane protein 2 (PRRT2) as the key research object.

## 2 MATERIALS AND METHODS

### 2.1 Animal

Forty‐five male Sprague‐Dawley (SD) rats weighing 190‐210 g were purchased from Beijing Vital River Laboratories (Beijing, China). Before any experimentation, all of the rats were allowed to have 1 week to adapt to the environment of the laboratory at the Animal Center of Shandong University. Throughout the whole experiment process, the laboratory temperature was kept at 20 ± 2°C, at a humidity of 45%‐65%, and on a 12 h light/12 h dark cycle. During the 1‐week adaptive phase, the rats were housed in groups and had free access to water and food. All of the experimental procedures involving the animals were previously approved by the Animals Care Committee of Shandong University, in line with the US National Institute of Health Guide for the Care and Use of Laboratory Animals and conforming to the ARRIVE guidelines.

### 2.2 Drugs and treatment groups

SA (Chengdu Must Bio‐technology, Sichuan, China) was dissolved in normal saline. Rats were randomly divided into three groups: the control group (C, n = 15), the CUMS group (M, n = 15) and the CUMS + Saikosaponin A group (50 mg/kg) (SA, n = 15). At the end of the fourth week, we conducted behavioural tests to prove that the stressors had worked. Then, the rats were intragastrically administrated with SA or saline once a day 30 min prior to stress exposure for four weeks. That is to say, rats in the SA group were intragastrically administrated with SA at a dose of 50 mg/kg, while the C group and M group were intragastrically treated with the same volume of normal saline.

### 2.3 CUMS procedure

To establish the depression animal model, we adopt the method of CUMS combined with separation as previously described with minor modifications,^23^ which is generally accepted and widely used. As is mentioned above, we had divided the experimental rats into three groups: the C, M, and SA (50 mg/kg) groups. Leaving the C group aside, the other two groups were subjected to eight kinds of stressors throughout the procedure. The stressors were as follows: fasting for 48 hours, water deprivation for 24 hours, physical restraint for 6 hours (from 9:00 to 15:00), 60°C cage tilt for 24 hours, cold swimming (at 4°C) for 5 minutes, tail pinch for 1.5 min (1 cm from the end of the tail), inversion of the light/dark cycle and damp sawdust for 24 hours. One stressor was applied daily at different times, and the same stressor cannot be exerted twice in one week in case the experimental animals adapted to these stressors. The CUMS procedure lasted for 8 weeks.

### 2.4 Bodyweight Measurement and Behavioural Testing

Before CUMS, we measured the experimental rat bodyweight as their initial weight (W1). Subsequently, the bodyweights were measured once a week during the whole CUMS process, recorded as W2, W3, W4, W5, W6, W7, W8 and W9. Then, we could draw the bodyweight growth curve and calculate the increase of bodyweight (W9 − W1). Behavioural tests were performed from the next day after eight weeks of CUMS exposure in sequence as follows.

#### 2.4.1 Open field test

The spontaneous exploratory behaviour was measured in the open field test (OFT), which was performed with minor modifications as described previously.^22^ In accordance with the reference, we made an open box (80 × 80 × 40 cm), which was painted with black inside, and the floor was divided into 25 equal lattices by white lines. At the beginning of the test, rats were placed individually in the centre of the open box and then left to explore freely for a 5‐min session. The horizontal locomotor activity (segments crossed with four paws) and vertical activity (number of rearings) were recorded. The total number of crossing and rearing was calculated and used as a measurable indicator of experimental animals’ spontaneous exploratory behaviour. The apparatus was cleaned with detergent prior to each test session to remove any olfactory cues.

#### 2.4.2 Sucrose preference test

All of the rats included in the blank control group were placed individually in the cages during the sucrose preference test (SPT). The entire SPT lasted for 4 days. The first two days were used to train experimental rats to drink sucrose water, that is, every rat was offered two bottles of 1% sucrose water for 24 hours. Then, one bottle of sucrose water was replaced by tap water. All of the rats were deprived of food and water on the third day. At 20:00 of the fourth day, each rat was provided with two bottles of water, one contained 400 mL sucrose water and the other 400 mL tap water. After 12 hours, the consumed volumes of sucrose solution and tap water were recorded. The sucrose preference, which is used as an index of anhedonia, was calculated using the following formula:

$$\text{Sp}=\text{sucrose consumption}÷\left(\text{water consumption}+\text{sucrose consumption}\right)\times 100\%$$

### 2.5 Measurement of 5‐HT, NE and DA levels in the hippocampus

All of the animals were deeply anesthetized with chloral hydrate and decapitated after the last behavioural test. Their brains were rapidly removed and put on ice. Adhered blood was rinsed by ice‐cold normal saline, and then, the hippocampi were dissected, frozen in liquid nitrogen and stored at −80°C. 5‐HT, NE and DA content in the hippocampus were determined by high‐performance liquid chromatography‐mass spectrometry (HPLC‐MS), using an Agilent 1200 series HPLC system (Agilent, USA). The chromatographic separation was carried out on an Agilent XDB C18 column (50 × 4.6 mm, 5 m; Waters) at 30°C. The samples were separated using a gradient mobile phase consisting of 5% methanol and 95% water at a flow rate of 0.2 mL/min. The injection volume was 10 μL. The mass spectrometer (Agilent, 6410B, USA) was operated in the positive ion electrospray mode with MRM. Data were acquired and processed using Agilent Mass Hunter software.

### 2.6 Screening the differentially expressed proteins in hippocampus by iTRAQ

#### 2.6.1 Sample preparation

In the study, we prepared eight sample pools: C1, C2, M1, M2, M3, SA1, SA2 and SA3. For the C group, the hippocampus of every four rats was mixed together as a sample pool; for the M group and the SA group, the hippocampus of every three rats was mixed together as a sample pool. Then, we add the right amount of SDT buffer, quartz sand and 1/4 inch ceramic beads (MP 6540‐424) into every sample pool. After that, the lysate was homogenized (24 × 2, 6.0 M/S, 60 s, twice) by MP homogenizer followed by sonication and then boiling for 15 minutes. After centrifugation at 14 000 g for 40 minutes, the supernatant was filtered with 0.22‐µm filters. The filtrate protein concentration was quantified with the BCA Protein Assay Kit (Bio‐Rad, USA). The sample was stored at −80°C.

#### 2.6.2 Preliminary experiment

Twenty micrograms of proteins for each sample were taken out and mixed with 5× loading buffer, followed by boiling for 10 minutes. Then, the proteins were separated on 12.5% SDS‐PAGE gel. Protein bands were visualized by Coomassie Blue R‐250 staining.

#### 2.6.3 FASP digestion and 8‐plex iTRAQ labelling

Thirty microlitres of protein solution from each sample was sequentially lysed, washed, blocked and digested, and finally, the peptides of each sample were desalted on C18 Cartridges (Empore^™^ SPE Cartridges C18 (standard density), bed ID 7 mm, volume 3 mL, Sigma), concentrated by vacuum centrifugation, and reconstituted in 40 µL of 0.1% (v/v) formic acid. The peptide content was estimated by UV light spectral density at 280 nm. Next, 100 μg peptide mixture of each sample was labelled with the eight iTRAQ reagents (termed here 113, 114, 115, 116, 117, 118, 119 and 121) using iTRAQ reagent according to the manufacturer's instructions (Applied Biosystems). The sample pool and their corresponding tags are shown in Figure 3A. Subsequently, the eight labelled samples were pooled, centrifuged and dried.

#### 2.6.4 HPLC and LC‐MS/MS analysis

The iTRAQ‐labelled peptide mixtures were separated using an Easy nLC Liquid Chromatograph (Thermo Scientific). The peptide mixture was loaded onto a reverse‐phase trap column (Thermo Scientific Acclaim PepMap100, 100 μm × 2 cm, nanoViper C18) connected to the C18 reversed‐phase analytical column (Thermo Scientific Easy Column, 10 cm long, 75 μm inner diameter, 3 μm resin), followed by a mobile phase elution with buffer A (0.1% formic acid) and buffer B (84% acetonitrile and 0.1% formic acid). Peptides were then eluted in a linear gradient with buffer B from 0% to 100% over 60 min at a flow rate of 300 nL/min. LC‐MS/MS analysis was performed on a Q Exactive mass spectrometer (Thermo Scientific) that was coupled to an Easy nLC (Proxeon Biosystems, now Thermo Fisher Scientific) for 60 min. The mass spectrometer was operated in positive ion mode. MS data were acquired using a data‐dependent top 10 method dynamically choosing the most abundant precursor ions from the survey scan (300‐1800 m/z) for HCD fragmentation. The instrument was run with peptide recognition mode enabled.

#### 2.6.5 Data analysis

A MASCOT engine (Matrix Science, London, UK; version 2.2) embedded into Proteome Discoverer was used to search for MS/MS spectra.

#### 2.6.6 Bioinformatics analysis

Gene Ontology (GO) annotation was used to annotate the target proteins, and the progress was roughly divided into four steps: blast, mapping, annotation and annotation augmentation. The Kyoto Encyclopedia of Genes and Genomes (KEGG) pathway annotation of target proteins was performed by using KEGG Annotation Automatic Server (KAAS) software. The FASTA protein sequences of differentially measured proteins were blasted against the online KEGG database to retrieve their KOs and were subsequently mapped to pathways in the KEGG. Then, the corresponding KEGG pathways were extracted.

### 2.7 Measurement Of PRRT2 Expression At tissue level

Western blot was performed to verify the protein expression level of PRRT2 in hippocampus. The Western blotting analysis was carried out as previously described^24^ with minor modifications. Briefly, the rat hippocampus tissues were homogenized in 500 μL ice‐cold RIPA lysis buffer (Beyotime, Shenzhen, Guangdong, China) using a tissue homogenizer, followed by sonication for 10 s and centrifugation (20 min, 15 000 rpm, 4°C). Then, the supernatants were collected. The protein concentration was measured with the BCA protein assay kit (Beyotime, Jiangsu, China). The protein (20 μg per lane) was separated by 10% SDS‐PAGE gels and was transferred to PVDF membranes (Millipore, Billerica, MA, USA). After blocking with 5% non‐fat dry milk at room temperature for 2 hours, the PVDF membranes were incubated with the appropriate primary antibodies overnight at 4°C: polyclonal rabbit anti‐PRRT2 antibody (1:500, ab167130; Abcam), polyclonal rabbit anti‐CPNE7 antibody (1:1000, ab111478; Abcam) or monoclonal mouse anti‐β‐tubulin antibody (1:5000, AC021; Abclonal). Following washing three times with TBST buffer and one time with TBS buffer, the blots were incubated with horseradish peroxidase‐labelled goat anti‐rabbit (mouse) IgG (1:5000; ZSGB‐BIO, Beijing, China) for 1 hour followed by three times washing with TBS. Finally, the antibody‐reactive bands were visualized by a Lumina Enhanced Chemiluminescent Kit (Millipore, Billerica, MA, USA) and a gel imaging system (Tanon Science & Technology Co., Ltd., China).

### 2.8 Measurement of PRRT2 expression at cell level

To further explore the relationship between SA and PRRT2 at cellular level, we used the corticosterone solution to stimulate PC12 cell, simulating the state of stress injury of nerve cells in patients with depression. Firstly, it is necessary to determine the optimal concentration of corticosterone. We use different concentrations of corticosterone solution (0, 50, 100, 200, 300, 400, 500 and 600 μmol/L)to simulate PC12 cells, respectively. After 24 hours of stimulation, the CCK8 assays were used to determine the effect of different concentrations of corticosterone on PC12 cell survival rate. Secondly, we need to determine the optimal concentration and pre‐treatment time of SA, We pre‐treated PC12 cells with different concentrations of SA solution (0.5, 1, 2, 4, 5, 6, 8 and 10 μmol/L), and the pre‐treatment time was, respectively, 0, 1, 2, 4 and 8 h. Similarly, the CCK8 assays were used again to determine the effect of different concentrations and pre‐treatment time of SA on PC12 cell survival rate. Thirdly, we use the optimal concentration SA to pretreat PC12 cell in appropriate time, after that the optimal concentration of corticosterone was added to PC12 cell. After 24 hours, we extracted cell proteins and measured the protein expression level of PRRT2 using western blot.

### 2.9 Data analysis

Statistical analysis was carried out using SPSS version 22.0. All of the data are expressed as mean ± SEM. The data were analysed in a one‐way ANOVA followed by the LSD test. The level of significance was set at *P* < .05.

## 3 RESULTS

### 3.1 CUMS depression model was successfully established and SA could reverse depressive behaviours

**Figure 1:**
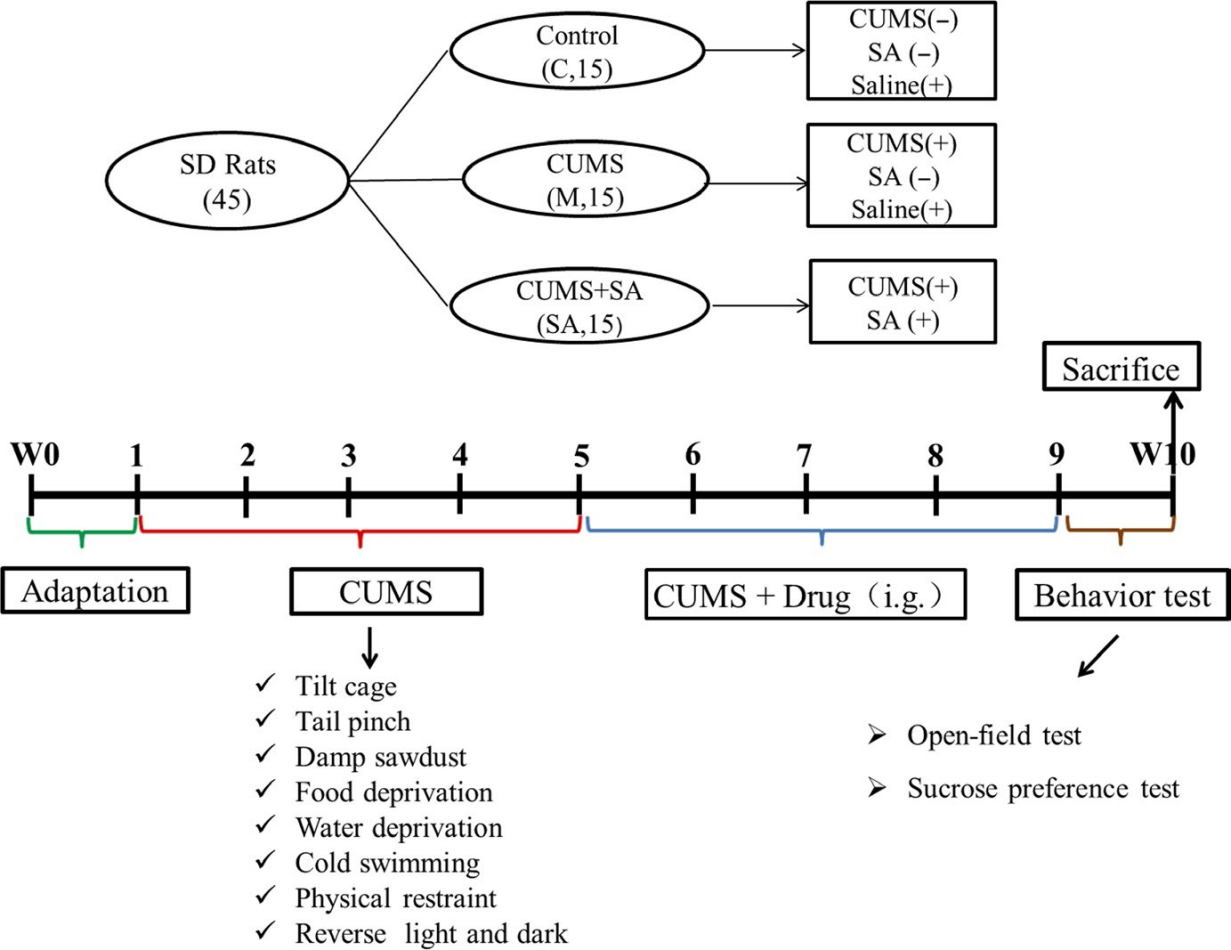
A, The details of experimental animals’ grouping and drug treatment. Forty‐five rats were divided into three groups: the C group, the M group and the SA group. The SA group was treated with SA at a dose of 50 mg/kg for 4 weeks, while the C and M groups were given the same volume (2 mL) of saline. B, Scheme of the CUMS protocol. The whole process lasted for 10 weeks: one week for adaptation, eight weeks for the CUMS procedure and one week for behaviour tests. After the last behaviour test, all of the rats were decollated and the tissue of the hippocampus was obtained

The method of 8‐week CUMS exposure combined with separation successfully established the rat depression model, resulting in significant behavioural changes. The experimental animals’ grouping and administration details are presented in Figure 1A. The whole process of the CUMS procedure is schematically presented in Figure 1B.

#### 3.1.1 SA reverted the diminished bodyweight gain induced by CUMS

**Figure 2:**
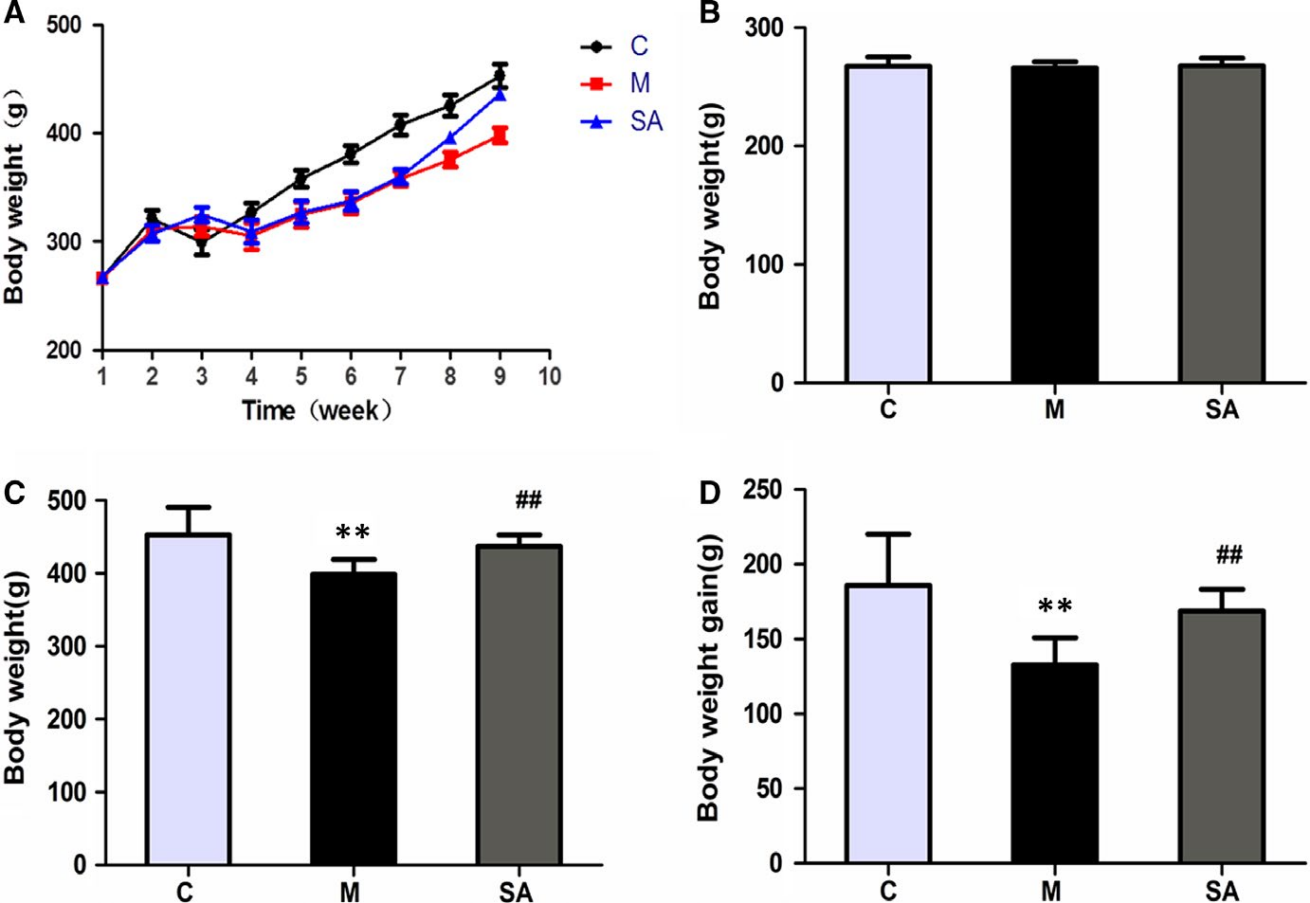
Effects of SA on experimental animal bodyweight. A, Weight growth curve during the whole process of building the depression model. With time, the bodyweight of each group gradually increased. The weight growth rate of rats in the M group was significantly lower than that of the C group, and the rate of the SA group was between those of the M group and the C group. B, Bodyweight of each group before CUMS procedure (W1). There was no significant difference between the groups (*P* > .05). C, The final bodyweight (W9) of each group. D, The increase of bodyweight relative to the initial weight (W9 − W1). SA (50 mg/kg) reversed the weight changes induced by CUMS exposure. All of the values are presented as means ± SEM. **P < .01 as compared with the C group. ##*P* < .01 as compared to the M group. Data was analysed in a one‐way ANOVA followed by the LSD test

Rat bodyweights were measured to assess the efficacy of CUMS and the antidepressant efficacy of SA. Figure 2A shows the weight growth curves of the three groups (C, M and SA) during the CUMS procedure. The initial bodyweight (W1), the last measured weight (W9) and the weight gain (W9 − W1) of the three groups are shown in Figure 2B‐D. These data indicate that rats suffering CUMS reveal a decreased bodyweight gain relative to the control group of animals (*P* < .01), while chronic administration of SA (50 mg/kg daily) significantly increased the bodyweight gain compared with the M group (*P* < .01).

#### 3.1.2 SA improved the animal performance in OFT

**Figure 3:**
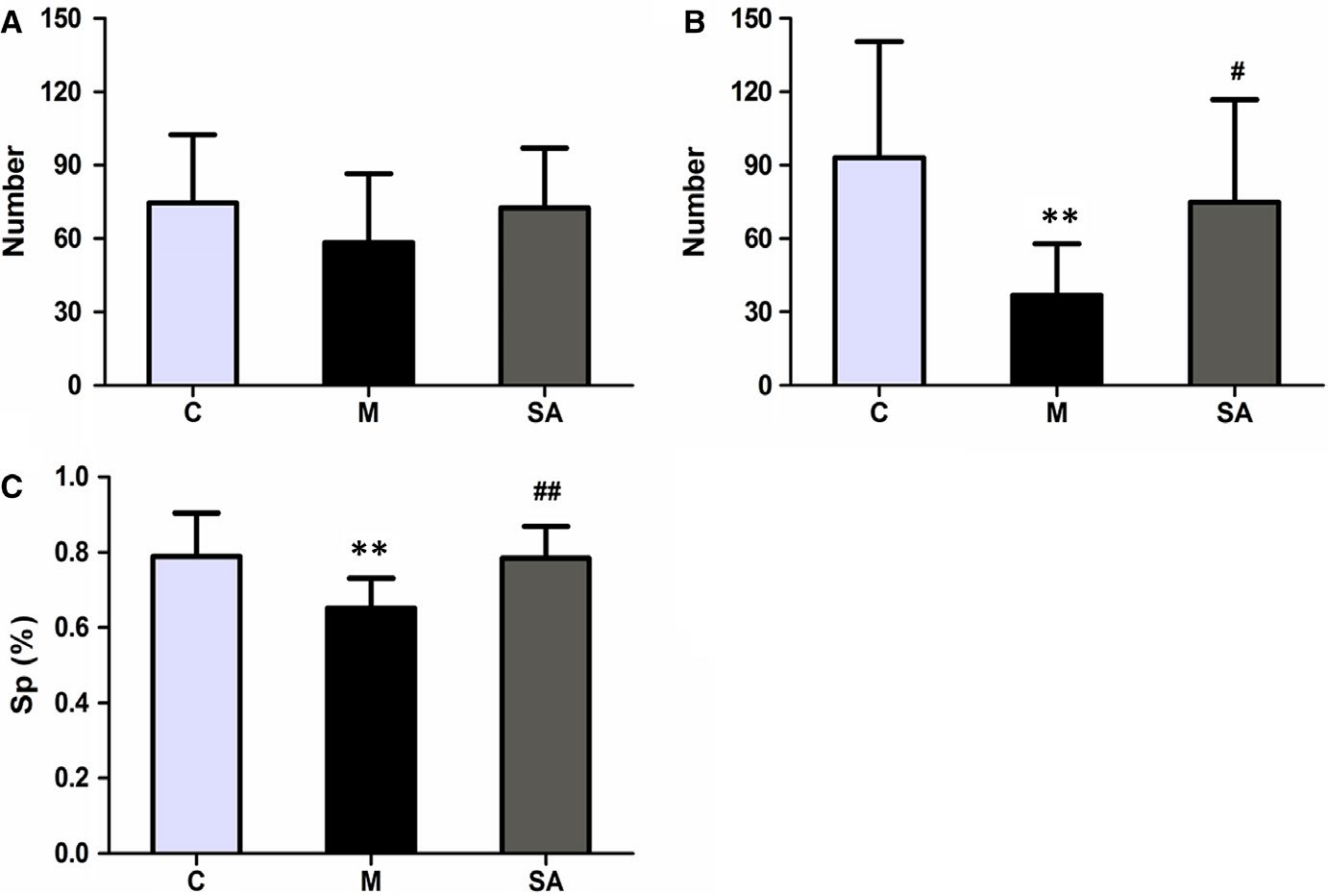
Effects of SA on behavioural tests. A and B, Effects of SA on the amount of locomotor activity (horizontal and vertical activity) in the OFT before and after the CUMS procedure. C, Effects of SA on the percentage of sucrose consumption. All of the values are presented as means ± SEM. **P < .01 as compared with the C group. #*P* < .05 as compared with the M group. ##*P* < .01 as compared with the M group in C. Data were analysed in a one‐way ANOVA followed by the LSD test

To explore the experimental animals’ spontaneous exploratory behaviour and anti‐anxiety effects, horizontal (number of crossings) and vertical (number of rearings) exploratory activity was measured in the OFT. Test results revealed that there was no significant difference among three groups in the total number of crossings and rearing before CUMS (Figure 3A). Figure 3B shows that CUMS exposure significantly reduced the total number of crossing and rearing (*P* < .01) in rats as compared to the C group. Chronic treatment with SA (50 mg/kg daily) significantly reversed the reduction as compared to the M group (*P* < .05).

#### 3.1.3 SA reverted the CUMS‐induced decrease in SP

The reduced consumption of sucrose solution is an indicator of anhedonia‐like behaviour. Figure 3C presented the effects of SA treatment on SPT performance in CUMS‐exposed rats. Eight‐week CUMS exposure significantly reduced the percentage of sucrose consumption as compared to the C group (*P* < .01), while chronic treatment with SA (50 mg/kg daily) significantly increased the percentage of sucrose consumption as compared to the M group (*P* < .01).

### 3.2 SA Increases the content of dopamine in hippocampus of rats exposed to CUMS

**Figure 4:**
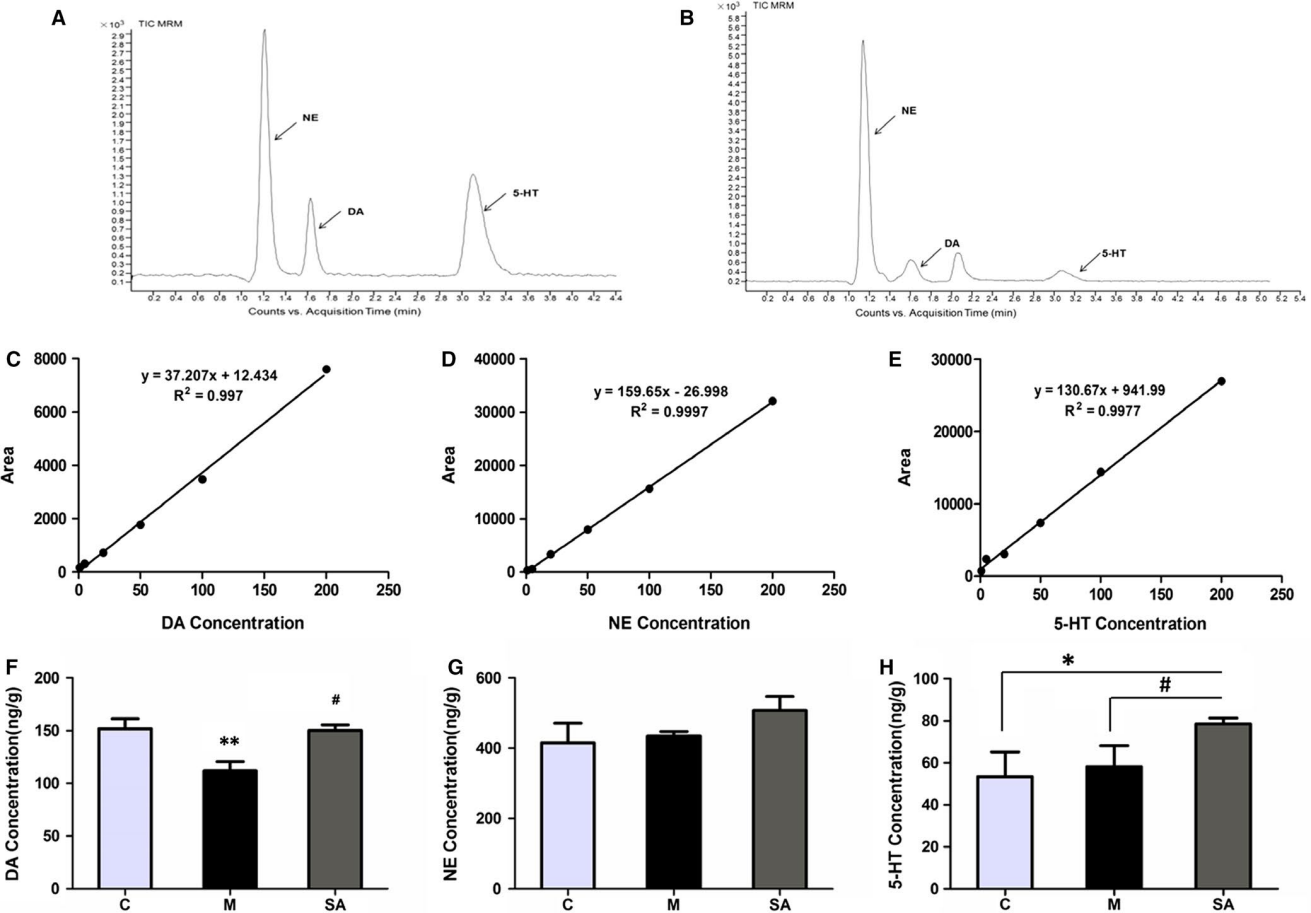
Effect of SA on 5‐HT, NE and DA levels in hippocampus. A, Chromatogram of monoamine neurotransmitter standard substance. B, Representative chromatogram of monoamine neurotransmitters from hippocampus of a control rat. C‐E, Standard curves of DA standard, NE standard and 5‐HT standard. F‐H, The concentration changes of monoamine neurotransmitters from the hippocampus of three rat groups. All of the values are presented as means ± SEM. **P < .01 as compared with C group. #*P* < .05 as compared with M group. Data were analysed in a one‐way ANOVA followed by the LSD test

The effect of SA on 5‐HT, NE and DA levels in the hippocampus of CUMS rats is shown in Figure 4, Tables 1 and 2. The data indicate that CUMS significantly reduced DA concentrations in the hippocampus of the M group compared with that in the C group (*P* < .01), while it had no significant influence on the 5‐HT and NE concentrations (*P* > .05). Administration of SA (50 mg/kg daily) was able to reverse the effects of CUMS on DA (*P* < .05).

**Table 1:** The linear relationship of DA, NE and 5‐HT standards

Analyte	Regression equation	r	Linear range (ng/mL)
DA	y = 37.207x + 12.434	0.997	5.00 ~ 446.64
NE	y = 159.65x − 26.998	0.9997	10.05 ~ 453.11
5‐HT	y = 130.67x + 941.99	0.9977	11.42 ~ 436.15

**Table 2:** Concentration of neurotransmitters in the hippocampus. The data indicate that CUMS significantly reduces DA concentrations in the hippocampus compared with that in control animals (*P* < .01), while it has no significant influence on the 5‐HT and NE concentrations (*P* > .05). Administration of SA (50 mg/kg daily) was able to counteract the effects of CUMS on DA (*P* < .05).

Analyte	C group	M group	SA group
DA	151.85 ± 9.32	112.07 ± 8.48	150.25 ± 5.10
NE	444.90 ± 32.17	434.60 ± 13.35	488.12 ± 29.87
5‐HT	58.30 ± 11.68	52.90 ± 4.35	78.51 ± 2.79

### 3.3 Proteomics screening results

#### 3.3.1 Differential expression of proteins in hippocampus screened by iTRAQ before and after the CUMS

**Figure 5:**
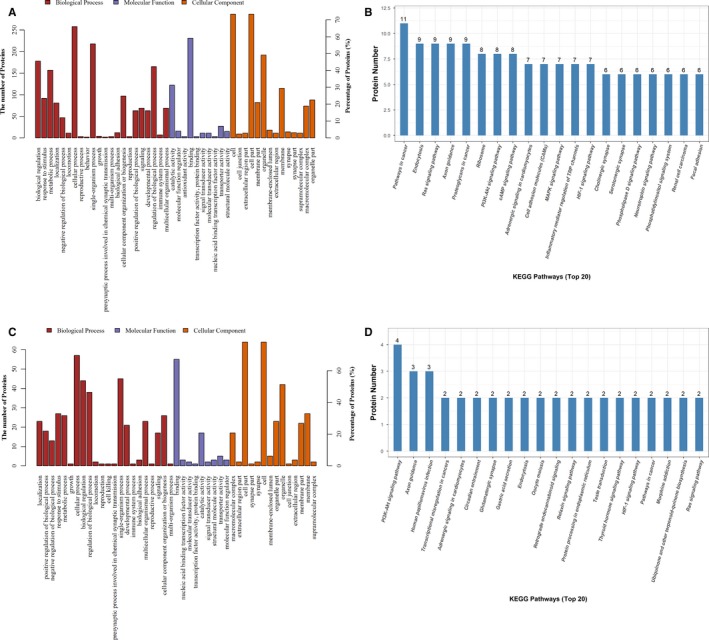
A, The analysis result of the GO annotation of differentially expressed proteins between the C and M groups. Biological process, molecular function and cellular component are marked, respectively, with the colours red, blue and orange. B, The statistical results of the top 20 KEGG pathways associated with the differentially expressed proteins between the C and M groups. C, GO annotation of differentially expressed proteins between the M and SA groups. D, The top 20 KEGG pathways associated with the differentially expressed proteins between the M and SA groups

To identify the proteins related to the CUMS stimulus, which may play a critical role in the pathogenetic process of depression, the quantitative proteomics technique iTRAQ was adopted in this study. Our results show that 4779 differentially expressed proteins were screened between the C group and the M group. According to the standard of ratio (M/C or SA/M) ≥ 1.2 and *P*‐value ≤ .05, 391 significantly differentially expressed proteins were selected (shown in Table S1). Among them, 123 proteins were up‐regulated compared with the C group (the ratio of M/C marked with red colour) and 268 proteins were down‐regulated (the ratio of M/C marked with blue colour). To obtain a full understanding of these proteins’ functions and interactions with each other, the GO annotation and KEGG pathway annotation were performed. The results of the GO annotation of these 391 proteins (Figure 5A) show that these proteins are involved in the composition of cell; organelles, membranes, macromolecular complexes and so on. In terms of molecular function, they mainly possess binding, catalytic and transporter activity. The biological process they mainly participate in are cellular processes; biological regulation processes, metabolic processes, responses to stimuli and so on. The result of the KEGG pathway annotation indicates that these 391 differentially expressed proteins are mostly involved in cancer, endocytosis, axon guidance and Ras signalling pathways. The top 20 pathways associated with the 391 proteins are shown in Figure 5B.

#### 3.3.2 Differentially Expressed Proteins in Hippocampus Regulated by SA

The same method was adopted to analyse the hippocampus proteome of rats exposed to CUMS and treated with SA or not. There were 4775 proteins screened out by iTRAQ between the M group and the SA group. Eighty‐two significantly differentially expressed proteins were selected from these 4775 proteins, based on the criteria of ratio (M/C or C/M) ≥ 1.2 and *P*‐value ≤ .05. The detailed information of these 82 proteins is shown in Table S2. Among the 82 proteins, 23 proteins were up‐regulated (the M/C ratio marked with red colour) while 59 of those were down‐regulated (the M/C ratio marked with blue colour). The results of the GO annotation and the KEGG pathway annotation are shown in Figure 5. The results of the GO annotation (Figure 5C) reveal that, in the aspect of cell components, the 82 proteins mostly participate in the composition of cell parts; organelles, membranes, synapses and so on. In the aspect of molecular function, they possess binding, catalytic, transporter and transcription factor activity. In the aspect of biological process, these proteins mainly take part in cellular processes, such as biological regulation, single‐organism process, responses to stimuli and metabolic process. The results of the KEGG pathway annotation demonstrate that the 82 differentially expressed proteins mostly take part in the PI3K‐Akt signalling pathway, axon guidance, circadian entrainment, glutamatergic synapses, endocytosis and morphine addiction signalling pathways. The detailed information of the top 20 pathways associated with the 82 proteins is shown in Figure 5D.

#### 3.3.3 Significantly differentially expressed proteins regulated by both CUMS and SA

By comparative analysis, 15 proteins were identified that not only were influenced by CUMS but were also influenced by SA. The specific information about these 15 proteins is presented in Table 3. Among these proteins, PNMA2 and CPNE7 were up‐regulated when rats were exposed to CUMS and chronic administration with SA (50 mg/kg daily) markedly decreased their expression levels. Proteins, including PRRT2, SART1, CaMKII, DENND4B, DOHH, FMN1, TMEM160, REM2, SNX18, PTER, LIMD2 and GIMAP8, were down‐regulated during the CUMS procedure, while chronic treatment with SA (50 mg/kg daily) significantly counteracted this change. The other three proteins (GIMAP8, REM2 and TMEM160) were not only down‐regulated during the CUMS procedure, but they were also down‐regulated by SA (50 mg/kg). These proteins, located in different parts of the cell, have different physiological functions and are involved in various signalling pathways.

**Table 3:** These 15 kinds of proteins had different expression levels both between groups M and C and between groups M and SA. Two proteins marked with red were up‐regulated during the CUMS procedure but down‐regulated by SA. Ten proteins, shown in blue, were down‐regulated during the CUMS procedure and were up‐regulated after the chronic administration of SA. The other three proteins were down‐regulated during the CUMS procedure and also after SA treatment; they are presented in green.

Number	Accession	Protein description	Abbreviation	M/C	SA/M
1	D4A068	Paraneoplastic Ma antigen 2	PNMA2	1.3976	0.7674
2	D3ZWR4	Copine VII	CPNE7	1.3813	0.7975
3	Q4KM31	LIM domain‐containing protein 2	LIMD2	0.8063	1.2457
4	Q63530	Phosphotriesterase‐related protein	PTER	0.7893	1.2074
5	D3ZZ38	Sorting nexin	SNX18	0.7156	1.3461
6	F1LR33	Phospholipid phosphatase‐related protein type 2	PIPPR2	0.6801	1.3065
7	D4A7C2	Formin 1	FMN1	0.6715	1.3144
8	Q5PPJ4	Deoxyhypusine hydroxylase	DOHH	0.6309	1.2002
9	F1M3F8	Calcium/calmodulin‐dependent protein kinase type II subunit gamma	CaMK2	0.5808	1.9329
10	D3ZFB6	Proline‐rich transmembrane protein 2	PRRT2	0.4972	1.4968
11	F1LTT7	DENN domain‐containing 4B	DENND4B	0.4120	1.3156
12	Q5XIW8	U4/U6.U5 tri‐snRNP–associated protein 1	SART1	0.3899	1.5335
13	Q4KLG2	GTPase IMAP family member 8	GIMAP8	0.8324	0.8195
14	A0JPL9	Rem2 protein	REM2	0.7018	0.8060
15	D3ZZU4	Putative uncharacterized protein	TMEM160	0.6852	0.8265

### 3.4 PRRT2 expression level changes in hippocampus

**Figure 6:**
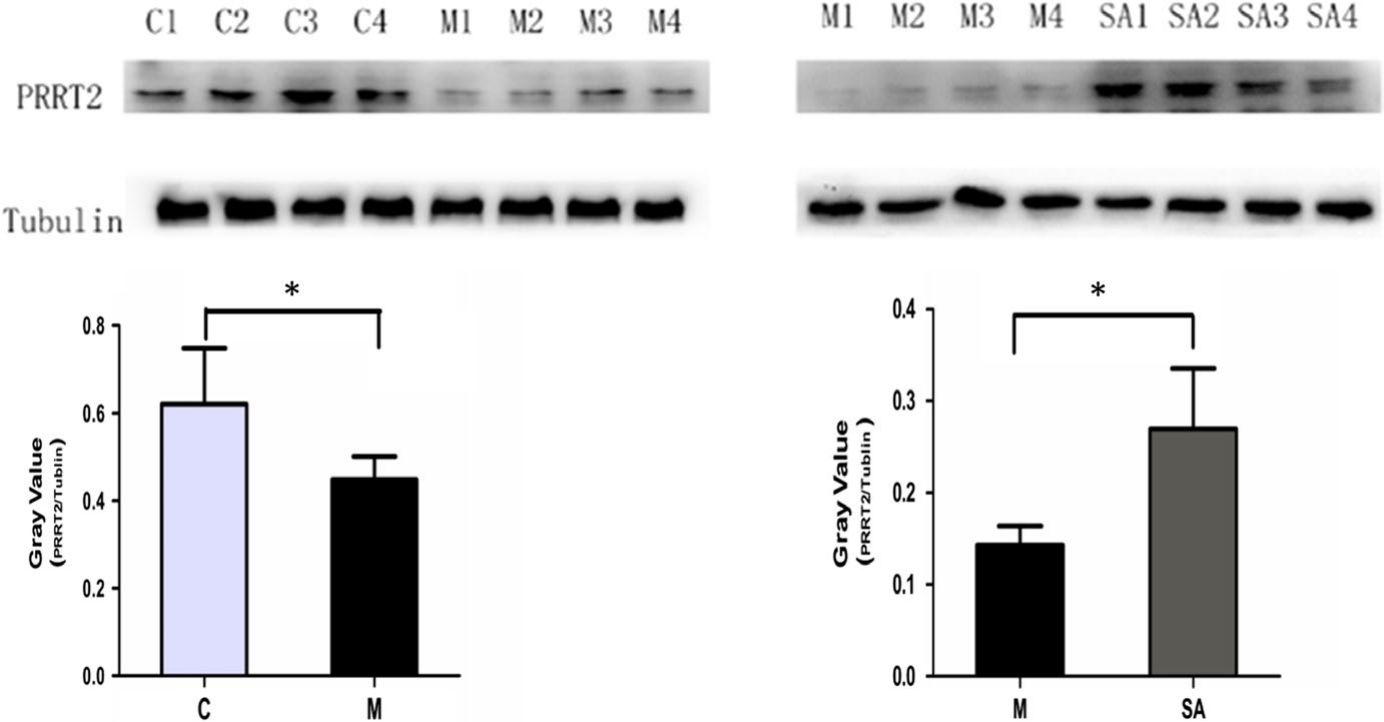
The expression level of PRRT2 in rat hippocampus. Normalized intensity bands of PRRT2 expression levels were presented as the means ± SEM

Western blot was performed to confirm the results of iTRAQ. As shown in Figure 6, the expression level of PRRT2 was indeed significantly down‐regulated by CUMS (*P* < .05), and 4‐week administration with SA (50 mg/kg daily) dramatically reversed the change (*P* < .05).

### 3.5 PRRT2 expression level changes in PC12 cell

**Figure 7:**
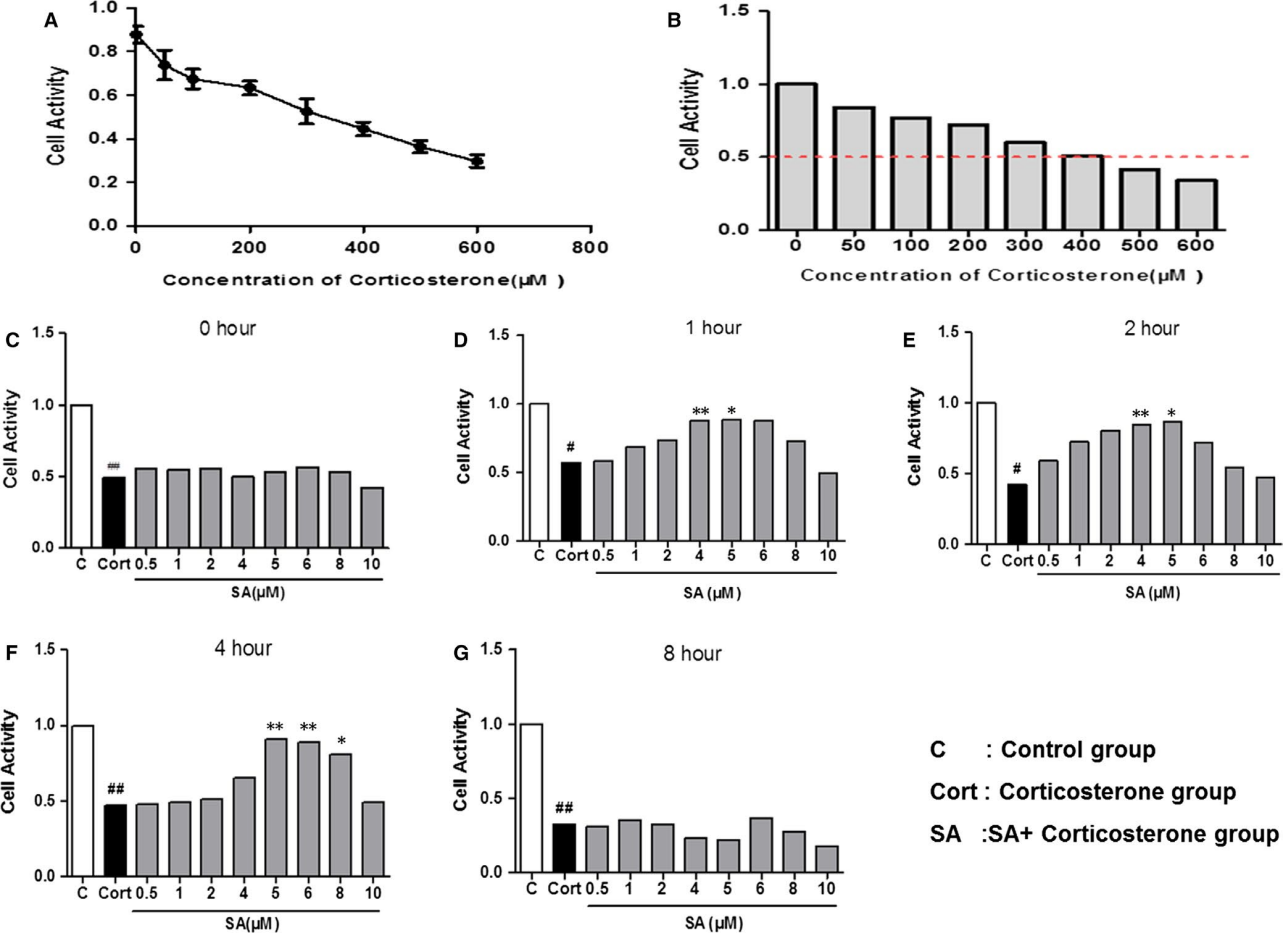
SA could reduce the neurotoxic effect of corticosterone on PC12 cells. A, With the increase of corticosterone concentration, the survival rate of PC12 cells decreased gradually. B, The survival rate of PC12 cells was 50% of the control group when the corticosterone concentration was 400 μmol/L. C, The effect of different concentrations of SA on the survival rate of PC12 cells when the pre‐treatment time of SA was 0 h, ##*P < *.01 compared with C group. D, The effect of different concentrations of SA on the survival rate of PC12 cells when the pre‐treatment time of SA was 1 h, #*P* < .05 compared with C group, **P < .01 compared with Cort group, *P < .05 compared with Cort group. E, The effect of different concentrations of SA on the survival rate of PC12 cells when the pre‐treatment time of SA was 2 h, #*P* < .05 compared with C group, **P < .01 compared with Cort group, *P < .05 compared with Cort group. F, The effect of different concentrations of SA on the survival rate of PC12 cells when the pre‐treatment time of SA was 4 h, ##*P* < .01 compared with C group, **P < .01 compared with Cort group, *P < .05 compared with Cort group. G, The effect of different concentrations of SA on the survival rate of PC12 cells when the pre‐treatment time of SA was 8 h, ##*P* < .01 compared with C group. Data were analysed in a one‐way ANOVA followed by the LSD test

**Figure 8:**
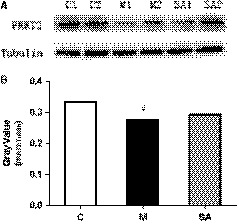
The expression levels of PRRT2 in PC12 cell were measured by Western blotting. 400 μmol/L corticosterone was able to significantly down‐regulated the expression of PRRT2 in PC12 cell, and 5 μmol/L SA had the trend of up‐regulating the expression of PRRT2, #*P* < .05 compared with C group. Data were analysed in a one‐way ANOVA followed by the LSD test

Results in vitro showed that 400 μmol/L corticosterone could significantly inhibit the proliferation of PC12 cell, causing a decrease of cell survival rate (Figure7A‐B).While 5 μmol/L SA(pre‐treat for 4 hours) could successfully reduce the inhibitory effect of corticosterone on cell proliferation and improve survival rate of PC12 cell (Figure7C‐G).The result of Western blot showed that 400 μmol/L corticosterone could significantly down‐regulated the expression level of PRRT2(*P* < .05), and 5 μmol/L SA (pre‐treat for 4 hours) could up‐regulate PRRT2 expression level to some extent (Figure8).

## 4 DISCUSSION

In the present study, the method of 8‐week CUMS combined with separation induced depressive‐like behaviours and caused a decrease of DA content in the hippocampus, as expected. Importantly, chronic administration of SA at a dose of 50 mg/kg could significantly alleviate depression symptoms and reverse the decrease of DA. PRRT2, one of the 15 kinds of differentially expressed proteins in the hippocampus, was proven to play a key role in the process of neurotransmitter release.^25^ In order to further explore the relationship among SA, the expression level of PRRT2, and the content changes of DA in hippocampus, we preliminarily investigated the biological function of PRRT2 at the tissue and cell level. And our results indicated that SA may affect DA content in hippocampal tissue by regulating the expression level of PRRT2. Thus, we believe that PRRT2 may be an important protein target for SA to exert antidepressant effects.

For the first time, adopting a quantitative proteomic technology—iTRAQ—we screened 15 kinds of differentially expressed proteins in the hippocampus between the rats with and without CUMS exposure and between the CUMS rats with and without SA treatment. To further clarify the functions and links among these proteins, GO annotation enrichment analysis and KEGG signalling pathway enrichment analysis were used. And the GO annotation enrichment analysis results show that these proteins were involved in the formation of cellular components, such as cell membrane, organelles, synapses and partial structures of synapses. They exert binding functions, catalytic activities, nucleic acid binding functions and transduction in terms of molecular function. In biological processes, it is mainly involved in regulating cellular processes, stress response processes, biological metabolic process, etc KEGG signalling pathway enrichment analysis results suggest that these differentially expressed proteins are mainly involved in the following signalling pathways: pathways in cancer, endocytosis pathways, axon guidance pathways, Ras signalling pathways, neurotrophin signalling pathways and PI3K‐Akt signalling pathways. As mentioned above, the pathogenesis of depression involves several systems, multiple proteins and a lot of small molecules. No single theory or signal pathway can fully elucidate its pathogenesis. Proteomics technology has made it possible for us to comprehensively explore the pathogenesis of depression. These differentially expressed proteins could be regulated by SA and may be potential drug targets for SA, which exerts antidepressant effects.

Our study suggested that the content of DA was, as expected, reduced when rats were subjected to CUMS, and the 4‐week administration of SA (50 mg/kg) counteracted this change. The content of 5‐HT and NE did not show a significant change between the C group and the M group or between the M group and the SA group. According to the results, we speculate that SA may exert antidepressant effects by increasing the DA content in the hippocampus. Therefore, it is necessary for us to understand the biological function and the transmission process of DA. According to existing studies, DA is regarded as the key regulator of reward mechanisms and drug addiction.^26^, ^27^ In recent years, however, basic and clinical studies have demonstrated that DA is also involved in the pathogenesis of depression.^28^ As is well known, the delivery process of typical neurotransmitters roughly includes the following steps: docking of synaptic vesicles on the presynaptic membrane, fusion of synaptic vesicles with the presynaptic membrane, the release of neurotransmitters and the clearance of neurotransmitters.^29^ Abnormal adjustment of any one of these steps will impede the transmission of neurotransmitters. In the present study, we mainly focused on the vesicle fusion and neurotransmitter release processes of DA. DA release is generally achieved by means of DA synaptic vesicle fusion with the presynaptic membrane of the DA neuron.^30^ The fusion and release processes of DA are regulated by many factors, such as DA synthesis, uptake and vesicular transport, as well as by Ca^2+^ homeostasis and regulatory exocytotic proteins.^29^ The main neurotransmitter release‐associated proteins include soluble N‐ethylmaleimide‐sensitive factor attachment protein receptor (SNARE) proteins 31, 32 and synaptotagmin 1 (Syt1).^33^, ^34^ The SNARE proteins are divided into t‐SNAREs and v‐SNAREs, according to their location. The protein VAMP2 is just one kind of v‐SNARE, which is located on the synaptic vesicle membrane. Synaptosomal‐associated protein 25 kD (SNAP‐25) and syntaxin 1 are members of t‐SNAREs, which are located on the presynaptic membrane.^35^, ^36^ A large number of studies have demonstrated that SNARE proteins are the core fusion machinery 37, 38 and Syt1 is the Ca^2+^ sensor that senses the concentration change of intracellular Ca^2+^ and binds to the Ca^2+^ ions.^39^, ^40^ When Ca^2+^‐bound Syt1 binds to SNAREs, the number of synaptic vesicle membrane fusion events will increase 50‐fold compared with membrane fusion mediated by SNAREs only.^30^ Summarizing all of the above findings, we conclude that SNAREs, Syt1 and Ca^2+^ play important roles in synaptic vesicle fusion and the release process. However, the mechanisms whereby these protein components orchestrate synchronized vesicle fusion and release processes in such a short time is still unclear at the molecular level.

Proline‐rich transmembrane protein 2, enriched in cerebral cortex, cerebellum, substantia nigra and hippocampus, is an uncharacterized protein that belongs to the PRRT superfamily. It consists of four exons and encodes a 340‐amino‐acid protein with two predicted transmembrane (TM) domains in the C‐terminal and one proline‐rich domain in the N‐terminal.^41^ PRRT2 is involved in a group of paroxysmal disorders, such as epilepsy, paroxysmal kinesigenic dyskinesia(PKD) and migraine 42, 43, but the PRRT2 function and pathogenic mechanisms remain largely obscure. A research published in Cell Research shows that PRRT2 is a presynaptic protein that interacts with components of the SNARE complex and down‐regulates its formation, and mutations in PRRT2 would induce PKD‐like phenotypes triggered by generalized seizures, hyperthermia or optogenetic stimulation of the cerebellum.^19^ According to the iTRAQ results in our study, the PRRT2 expression level was down‐regulated when rats were subjected to CUMS, while chronic treatment of SA (50 mg/kg) could counteract the change significantly. To our delight, a recent study performed by Pierluigi Valente and his colleagues indicated that PRRT2 was enriched in presynaptic terminals and its silencing decreases the number of synapses and increases the number of docked synaptic vesicles at rest. Also, using the method of co‐immunoprecipitation, they proved that PRRT2 interacts with Ca^2+^ sensors Syt1 and a number of SNARE proteins, including VAMP2, SNAP‐25 and syntaxin 1. The interactions with the SNARE proteins suggest that PRRT2 acts as a catalyst in the fusion processes, and the interactions with Syt1 indicate that PRRT2 participates in the regulation of the Ca^2+^ sensing apparatus for fast synchronous release.^25^, ^44^ Therefore, we speculate that PRRT2 orchestrates SNAREs and Syt1 in synaptic vesicle fusion and the release process. Without it, the Ca^2+^‐dependent neurotransmitter release process would be strongly impaired. Also, our proteomics results show that PRRT2 levels in rats in the M group were significantly decreased, while the chronic treatment of SA inhibited this decrease in PRRT2 expression levels. Consistent with these results, the DA content in the M group was reduced and the reduction was counteracted by SA treatment. Based on all of these conclusions and the results of our study, we speculate that PRRT2 serves as a regulatory factor in the fusion and release process of DA by orchestrating SNAREs and the Ca^2+^ sensor Syt1. Therefore, the chronic administration of SA would up‐regulate the expression level of PRRT2, and then, the increased PRRT2 level would up‐regulate the content of DA by mediating the fusion and release process of DA. Of course, the concrete interaction mechanism between SA and PRRT2 needs to be further explored.

**Figure 9:**
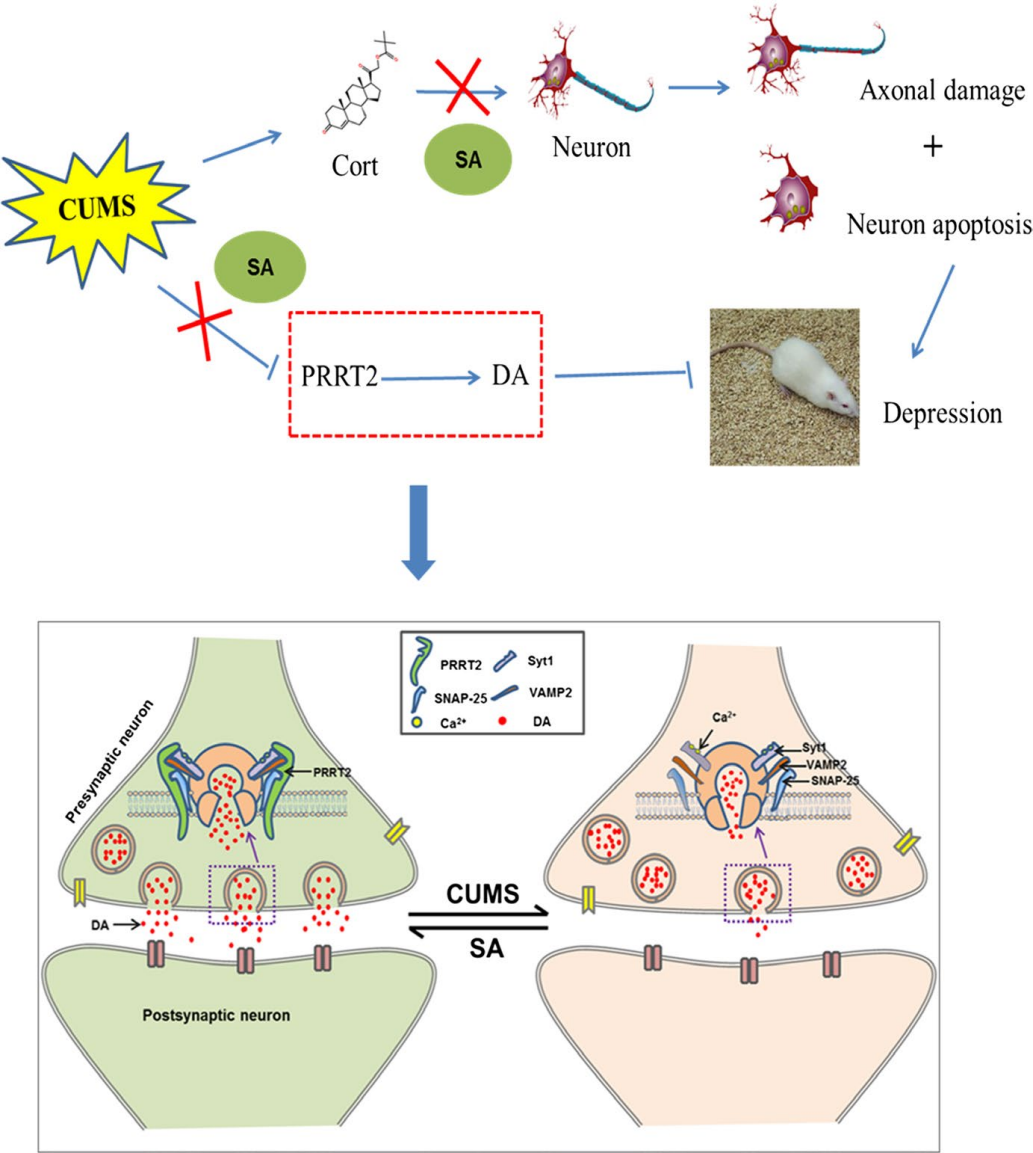
A, The mechanism diagram of the present study. Our study indicated that SA could significantly relieve depressive symptoms of CUMS rats. On one hand, SA could up‐regulate the expression of protein PRRT2 and increase the content of DA in hippocampus. On the other hand, SA can reduce the toxic effects of excessive corticosterone on nerve cells. Therefore, PRRT2 is expected to be a potential target protein for SA to exert anti‐depressive effects. B, PRRT2 played a key role in DA release process. PRRT2 is mainly located in presynaptic terminals of neurons. It acts as a catalyst and a regulator in the fusion and Ca2^+^‐sensing apparatus for fast synchronous release processes by interacting with SNARE proteins, including VAMP2, SNAP‐25 and the Ca2^+^ sensor Syt1. PRRT2 of rats subjected to the CUMS procedure was down‐regulated and the proteins Syt1, VAMP2 and SNAP‐25 could not interact well with each other. As a result, the release probability and amount of released DA were reduced in the hippocampus. Chronic administration of SA (50 mg/kg) could up‐regulate the PRRT2 expression level and then increase the release probability and the amount of released DA

In conclusion, the results of the present study suggest that the 4‐week administration of SA significantly increases the hippocampal content of DA. Moreover, we confirmed that the protein PRRT2, one of 15 differential expressed proteins, plays a crucial role in CUMS‐induced stress. The possible interaction mechanism between CUMS, SA, DA and PRRT2 was shown in Figure 9. The identification of PRRT2 and other differentially expressed proteins will provide us with novel candidate targets in the study of the anti‐depression mechanism of SA.

## CONFLICT OF INTEREST

The authors confirm that there are no conflicts of interest.

## AUTHORS' CONTRIBUTIONS

Shilian Liu, Kuanxiao Tang, Zhaoyu Qin and Juanjuan Guo designed the research study; Juanjuan Guo and Feng Zhang performed the research; Jifang Gao, Xinyuan Guan and Beiyun Liu contributed to the preparation of rat depression model; and Juanjuan Guo analysed the data and wrote the paper.

## Data Availability

The data that support the findings of this study are available from the corresponding author upon reasonable request.
